# Overcoming Anatomical Constraints: A Case of Successful Endovascular Repair in a High-Risk Patient With a Short, Angulated Abdominal Aortic Aneurysm

**DOI:** 10.7759/cureus.78972

**Published:** 2025-02-13

**Authors:** Le Duc Tin, Lam Van Nut, Nguyen Hoang Duc, Amjad S AlMosa, Nguyen Tien Huy

**Affiliations:** 1 Vascular Surgery, Cho Ray hospital, Ho Chi Minh, VNM; 2 School of Medicine, Hanoi Medical University, Ha Noi, VNM; 3 School of Medicine, Imam Abdulrahman Bin Faisal University, Dammam, SAU; 4 Institute of Research and Development, Duy Tan University, Da Nang, VNM

**Keywords:** abdominal aortic aneurysm, aneurysm neck characteristics, chimney technique, endovascular aortic repair, short aortic neck

## Abstract

Endovascular aneurysm repair (EVAR) for abdominal aortic aneurysms (AAAs) is often limited by anatomical factors such as short necks (less than 15 mm) and significant angulation (over 60 degrees), making EVAR unsuitable. We present a case of a 60-year-old man with multiple comorbidities and a short, markedly angulated AAA neck. Despite being deemed unsuitable for EVAR, the patient underwent successful EVAR with the chimney technique. At the one-month follow-up, the patient had no complications, stable renal function, and no detected endoleak or other complications on routine CT scans. Cardiac tests were normal. This case challenges traditional contraindications and highlights the potential for EVAR in challenging anatomical situations. Incorporating the chimney technique and reinforcing the proximal neck can prevent complications, while future research should focus on tailored EVAR strategies addressing individual needs and anatomical challenges.

## Introduction

Endovascular aneurysm repair (EVAR) is a minimally invasive procedure that has revolutionized the management of abdominal aortic aneurysms (AAAs), especially in high-risk patients. The procedure involves deploying a stent graft within the aorta to strengthen the weakened vessel wall and prevent rupture. The success of EVAR is influenced by several factors, including the anatomical characteristics of the aortic neck, where the stent graft is anchored [1]. Challenging aortic neck features, such as short length and significant angulation, have traditionally been considered barriers to EVAR. These challenges include achieving a neck length of 10-15 mm for secure graft sealing and ensuring an appropriate diameter and favorable angulation for proper stent graft placement and fixation. Once the angle exceeds 60 degrees, technical challenges and the risk of complications significantly increase, often requiring alternative treatment approaches, such as open surgery [2,3].

This report presents a successful case of using combined EVAR with an individualized approach in a patient with multiple comorbidities and an infrarenal AAA characterized by a very short and significantly angulated abdominal aortic neck. This case illustrates how innovative approaches can address traditional limitations, extending the applicability of endovascular interventions and potentially reducing the reliance on higher-risk open surgeries. It underscores the importance of exploring and advancing endovascular techniques to expand treatment options for challenging AAAs while carefully considering aortic neck characteristics and angulation limitations to ensure successful outcomes. 

## Case presentation

A 60-year-old male patient with a history of an infrarenal AAA presented with constant, dull abdominal and back pain that neither radiated nor worsened with movement. The AAA was initially detected two years ago via an abdominal CT scan, measuring 42 mm in diameter, and had since progressed to 53 mm. Although surgical intervention was recommended, the patient declined due to personal reasons and concerns about surgical risks. His medical history includes a previous myocardial infarction, five coronary bypass surgeries, hypertension, type 2 diabetes, and stage III chronic renal failure. He has been compliant with prescribed medications and medical advice. 

**Figure 1:**
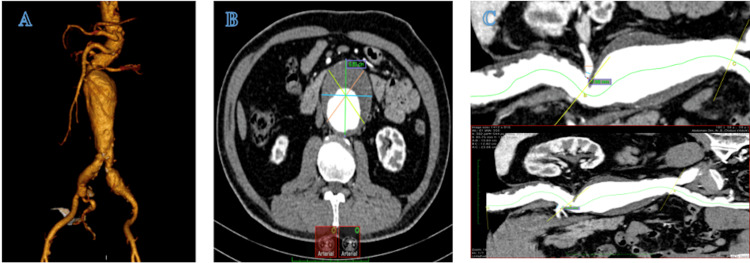
CT scan prior to the procedure showing the anatomical characteristics of the abdominal aortic aneurysm A) Significant short neck and angled aortic neck. B) Largest horizontal diameter of the abdominal aortic aneurysm. C) Stenosis observed in the left renal artery and short length of the right renal artery.

On admission, the patient had stable vital signs, including a pulse rate of 78 beats per minute and a blood pressure of 140/85 mmHg. Skin turgor was normal. Physical examination revealed a palpable mass to the left of the umbilicus, characterized by pulsation and tenderness upon pressure. A CT scan confirmed an infrarenal AAA measuring 68 mm in diameter. The aortic neck demonstrated an angulation of approximately 65 degrees, with a length of 15 mm from the right renal artery ostium. Posterior to the ostium, the neck was bifurcated into two branches extending to the upper and lower poles of the right kidney. The left renal artery ostium was located in close proximity to the aneurysm, with a neck length of approximately 5 mm (Figure 1).

Other measurements obtained from the CT scan for the planned endovascular procedure included a length of approximately 128 mm from the left renal artery to the aorta-iliac artery junction, a total length of about 190 mm from the lower kidney pole to the common iliac arteries (CIAs), right and left CIA diameters of 14 mm and 15 mm, respectively, and a common femoral artery diameter of approximately 9 mm. ECG showed normal sinus rhythm with evidence of a prior inferior wall myocardial infarction. Echocardiography revealed an ejection fraction of 54%, with no regional wall motion abnormalities. Laboratory tests indicated preserved renal function, normal blood counts, and well-controlled blood glucose levels. 

**Figure 2:**
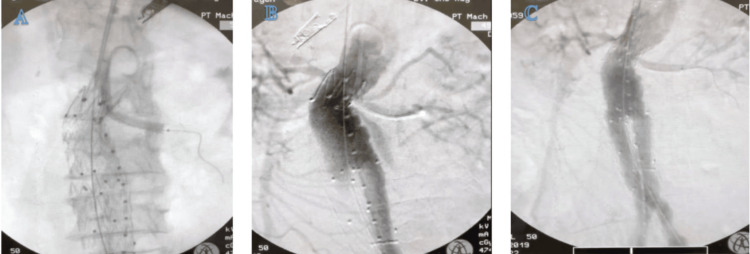
Completion of the aortogram obtained after the procedure, illustrating the successful outcome of the intervention A) Proximal landing zone and chimney technique utilized for the left renal artery. B) Detection of a type 1A endoleak. C) Resolution of post-EVAR endoleak following the placement of an additional cuff at the proximal neck of the aneurysm.

Given the patient's high-risk profile and complex aortic aneurysm, an EVAR combined with the chimney technique was performed on the left renal artery branch. After a thorough consultation, the patient provided informed consent, fully understanding the procedure, its benefits, potential risks, and alternative options. Local anesthesia with 2% lidocaine was administered, and a bolus of 3000 IU of heparin was given before the intervention. Access to the aneurysm was obtained by inserting a needle into both common femoral arteries, followed by placement of a 7 Fr sheath. Additionally, a needle was inserted into the right brachial artery, and a 6 Fr sheath was placed. The sheaths in the left and right femoral arteries were then exchanged for 20 Fr and 16 Fr long sheaths, respectively. A 0.035" soft guidewire, accompanied by a 5 Fr pigtail catheter, was introduced through the right femoral artery, advanced to the thoracic aortic arch, and then replaced with a 0.035" stiff guidewire, which was fixed in place. The 0.035" soft guidewire with a 5 Fr pigtail catheter was inserted from the left femoral artery to the abdominal aorta, advancing to identify the bilateral renal arteries accurately. The pigtail was then secured just above the celiac artery. With catheter support, a soft 0.035" guidewire was inserted through the right femoral artery to access the left renal artery. It was then replaced with a stiff 0.035" guidewire, advanced to the left renal artery, and stabilized. A 5x60 mm balloon was inflated to open the narrowed left renal artery, and a 6x58 mm covered stent was delivered to the left renal artery. The orifice of the bilateral renal arteries was confirmed after inserting the main body of the stent (25x14x103 mm) from the left femoral artery into the proximal landing zone, just below the right renal artery (Figure 2A). The projection angle was adjusted for clarity to highlight the right renal artery. Simultaneous deployment of the covered stent graft for the left renal artery and the main body of the stent graft was carried out, extending to the end of the right limb stent graft. Gate cannulation was performed from the right femoral artery access site for the right limb stent graft. After confirming the length of the stent graft, a 16x16x156 mm stent graft was deployed to the end of the right CIA. The left limb of the main body of the stent graft was expanded, and after confirming the length of the stent graft, a 16x16x156 mm stent graft was deployed to the end of the left CIA. After completing the aortogram, a type 1A endoleak was observed. To address this, an additional cuff was implanted at the proximal neck of the aneurysm. A subsequent completion aortogram confirmed the absence of any post-EVAR endoleak and demonstrated the patency of all graft components (Figures 2B, 2C).

**Figure 3:**
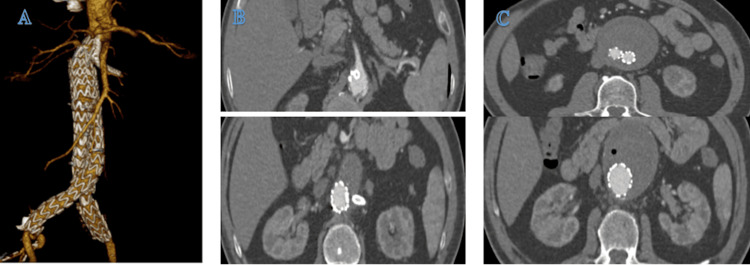
CT scan taken at the one-month follow-up appointment, demonstrating the post-procedure status of the abdominal aortic aneurysm A) Post-intervention status. B-C) Position of the chimney branch and stent-graft positioned without any observed endoleaks.

The sheaths were withdrawn from the puncture sites, using two ProGlide (Abbott Vascular, Santa Clara, California, USA) closures for each side of the femoral artery and one ProGlide closure for the right brachial artery. A compression bandage was applied over the needle puncture sites. Postoperatively, the patient's pulse, blood pressure, urine output, heart function, and kidney function remained stable, showing no significant changes compared to pre-surgery. The patient was discharged after a three-day hospital stay. At the one-month post-intervention follow-up, the patient remained asymptomatic, and a follow-up CT scan revealed no endoleak or other complications, confirming the procedure's success (Figure 3).

## Discussion

A study found that, compared to EVAR, open repair was associated with higher odds of 30-day mortality and perioperative complications. Still, it also demonstrated lower rates of 6-year mortality, rupture, and reintervention [4]. Besides, the treatment of AAAs remains controversial, particularly in patients at high physiologic risk for open repair and high anatomic risk for endovascular repair. However, EVAR has become widely used for treating infrarenal aortic aneurysms. Notably, "chimney" grafts have been proposed as a viable endovascular option for aneurysms involving critical side branches, such as the renal and superior mesenteric arteries [5]. Additionally, one study demonstrated that, in physiologically challenged patients at higher anatomic risk (e.g., those with a short or angulated neck), perioperative mortality rates for EVAR were similar to those of patients at lower risk [6]. In this case, the patient had multiple comorbidities and an infrarenal AAA with a very short and significantly angulated abdominal aortic neck. The open surgery required extensive clamping, reinsertion of the left renal artery, and replacement of the AAA with a Y-shaped prosthesis, all of which contributed to a prolonged operation time. This extended procedure increased the risks of myocardial infarction, heart failure, and progressive renal failure for the patient.

Considering these factors, our patient underwent endovascular intervention due to its advantages, including local anesthesia and faster postoperative recovery. However, the patient presented with challenging anatomical features, such as a short and significantly angulated aortic neck, along with severe renal artery stenosis. These features heightened the risk of complications, including endoleak and stent-graft displacement [1]. As a result, a conventional EVAR approach alone was deemed insufficient to address the complex anatomy. To overcome these challenges, a more tailored and strategic approach was implemented.

The chimney technique is a valuable approach for managing complex aortic aneurysms involving branch vessels, such as the renal arteries. Its key advantage lies in preserving blood flow to branch vessels while enabling effective endovascular repair of the aneurysm [7-9]. In this case, the patient's aneurysm was located near the renal arteries and featured a short, angulated neck. To address these challenges, the chimney technique was employed to accommodate the left renal artery. However, the right renal artery's early branching, approximately 5 mm from its root, made it unsuitable for the conventional chimney approach. As a result, the proximal landing zone had to be positioned below the right renal artery. To enhance stability and reduce the risk of complications, such as endoleak and stent-graft displacement, the use of EndoAnchors (Medtronic, Minneapolis, Minnesota, USA) is generally considered. However, in this case, the significantly short and angulated proximal aortic neck rendered EndoAnchors unsuitable. Instead, a proximal balloon-expandable stent was employed to reinforce the proximal neck area, effectively addressing its short length and complex angulation. Evidence suggests that using prophylactic adjunctive balloon-expandable stents may reduce the incidence of secondary interventions related to hostile neck anatomy when used in conjunction with EVAR [10].

At the one-month post-intervention follow-up, the patient presented as asymptomatic and complication-free, demonstrating the effectiveness of this approach. This method proved particularly advantageous for managing cases with significantly short and angulated necks, combined with multiple high-risk factors that precluded open surgery or conventional EVAR. The successful outcome of this case underscores the exceptional flexibility and adaptability of endovascular techniques in the treatment of complex aortic aneurysms.

## Conclusions

This case report highlights the significance of a personalized approach in managing complex AAAs in high-risk patients. Anatomical challenges, such as short necks, angulations exceeding 60 degrees, or unfavorable involvement of both renal arteries, often complicate the feasibility of relying solely on EVAR. Future research should prioritize developing tailored EVAR strategies that effectively address each patient's unique needs and anatomical complexities, as well as assessing long-term outcomes in a larger cohort. 
